# Eosinophil differentiation in the bone marrow is promoted by protein tyrosine phosphatase SHP2

**DOI:** 10.1038/cddis.2016.74

**Published:** 2016-04-07

**Authors:** L-x Xia, W Hua, Y Jin, B-p Tian, Z-w Qiu, C Zhang, L-q Che, H-b Zhou, Y-f Wu, H-q Huang, F Lan, Y-h Ke, J J Lee, W Li, S-m Ying, Z-h Chen, H-h Shen

**Affiliations:** 1 Department of Respiratory and Critical Care Medicine, Second Affiliated Hospital, Zhejiang University School of Medicine, Hangzhou, Zhejiang 310009, China; 2 Department of Pathology and Pathophysiology, Zhejiang University School of Medicine, Hangzhou, Zhejiang 310058, China; 3 Division of Pulmonary Medicine and Hematology and Oncology, Department of Biochemistry and Molecular Biology, Mayo Clinic Arizona, Scottsdale 85259, Arizona; 4 The State Key Laboratory of Respiratory Diseases, Guangzhou, Guangdong 510120, China

## Abstract

SHP2 participates in multiple signaling events by mediating T-cell development and function, and regulates cytokine-dependent granulopoiesis. To explore whether and how SHP2 can regulate bone-marrow eosinophil differentiation, we investigate the contribution of SHP2 in the bone-marrow eosinophil development in allergic mice. Blockade of SHP2 function by SHP2 inhibitor PHPS-1 or conditional shp2 knockdown by adenovirus-inhibited bone-marrow-derived eosinophil differentiation *in vitro*, with no detectable effects on the apoptosis of eosinophils. Furthermore, SHP2 induced eosinophil differentiation via regulation of the extracellular signal-regulated kinase pathway. Myeloid shp2 conditional knockout mice (*LysM*^*cre*^*shp2*^*flox/flox*^) failed to induce eosinophilia as well as airway hyper-responsiveness. The SHP2 inhibitor PHPS-1 also alleviated eosinophilic airway inflammation and airway hyper-responsiveness, accompanied by significantly reduced levels of systemic eosinophils and eosinophil lineage-committed progenitors in allergic mice. We demonstrate that inhibition of eosinophil development is SHP2-dependent and SHP2 is sufficient to promote eosinophil formation *in vivo*. Our data reveal SHP2 as a critical regulator of eosinophil differentiation, and inhibition of SHP2 specifically in myeloid cells alleviates allergic airway inflammation.

Asthma is a chronic airway disease characterized by reversible hyper-reactivity and progressive airway inflammation, especially infiltration of eosinophils into the airway. Eosinophils appear to be the key effector cells in asthma, and there is a positive correlation between increased numbers and activation of eosinophils and the severity of asthma.^1, 2, 3^ Increased numbers of eosinophils have also been found in the bone marrow of patients with atopic asthma.^4^ Eosinophils develop in the bone marrow, exit to the bloodstream and enter lung tissue in response to pro-inflammatory mediators such as eotaxin.^5, 6, 7^

Eosinophils develop from granulocyte/monocyte progenitors (GMPs) through intermediate eosinophil lineage-committed progenitors (EoPs) in mice,^8^ whereas human EoPs are derived from common myeloid progenitors or their upstream multipotent progenitors.^9^ Expression of the IL-5 receptor on EoPs is a result of commitment of GMPs to the eosinophil lineage, which means that IL-5 supports eosinophil development from EoPs rather than instructing GMPs to commit to the eosinophil lineage.^8^

The protein tyrosine phosphatase SHP2 is a ubiquitously expressed intracellular enzyme that contains two Src homology 2 domains and one catalytic protein tyrosine phosphatase domain.^10, 11, 12^ SHP2 integrates multiple signaling events and mediates a variety of physiological functions.^13, 14, 15^ Studies have shown that SHP2 participates in multiple signaling events by mediating T-cell development and function, and stimulating CEBPA gene expression to regulate cytokine-dependent granulopoiesis.^16, 17^ Normal SHP2 function is critical for the initial step of embryonic stem (ES) cell differentiation to mesoderm and to hemangioblasts. It acts within the LIF-gp130-Stat3 pathway to keep a proper balance of ES cell differentiation, pluripotency and apoptosis, thereby maintaining a functional hematopoietic stem cell/progenitor pool.^13, 18, 19, 20^ Pazdrak *et al.*^21, 22, 23^ demonstrated that the physical association of SHP2 with the phosphorylated *α* common chain of the IL-5 receptor (IL-5*α*cR) and Grb2, and its early activation, are required for coupling of the receptor to the Ras-Raf-MAP/Erk2 pathway and for the prevention of eosinophil death by IL-5, and shp2 may also act as both a positive effector to downstream GM-CSF- and ICAM-1-dependent ERK1/2 activation in human eosinophils.

In a previous study, we have shown that specific deletion of *shp2* expression in mouse airway epithelia reduces TGF-*β*1 production and attenuates allergic airway remodeling.^24^ However, as SHP2 is expressed ubiquitously, it is of interest to know its possible roles in other cells or tissues during the development of asthma. As eosinophils are crucial for the pathogenesis of asthma, it is important to understand how eosinophil differentiation is regulated. Therefore, we investigated whether and how SHP2 affects eosinophil development. By using the SHP2 inhibitor PHPS-1 and specifically deleting *shp2* in myeloid cells, we found that inactivation or loss of SHP2 in these cells decreased the level of eosinophil recruitment to the airway, resulting in alleviation of lung inflammation and reduction of airway hyper-responsiveness (AHR), which were most likely through a direct inhibition of eosinophil differentiation. These results suggest that SHP2 may be a key regulator of eosinophil differentiation and thus can serve as a potential therapeutic target for the treatment of asthma.

## Results

### SHP2 is required for eosinophil differentiation *in vitro* without influence on the apoptosis of eosinophils

**Figure 1:**
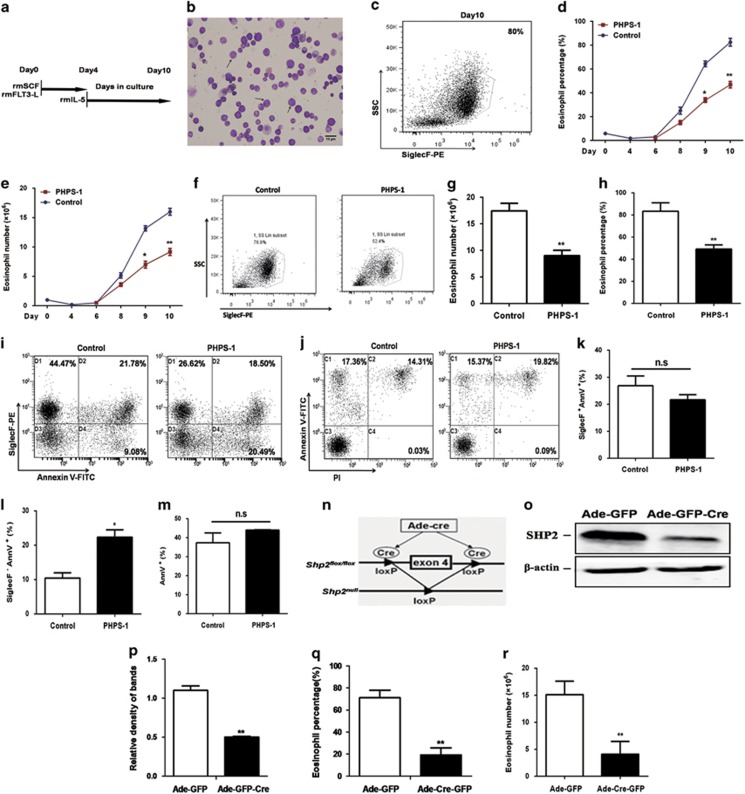
SHP2 knockdown inhibits bmEo differentiation *in vitro*, without influencing apoptosis. (**a**) *In vitro* culture of mouse bmEos following differentiation with rmSCF, rmFLT3-L and rmIL-5 as indicated. (**b**) Micrographs (× 400) of bmEos from WT mice on day 10, showing a stained Cytospin. Images were taken with an Olympus BX51 microscope (× 4/0.3 NA objective equipped with a mounted Olympus DP70 digital camera (Tokyo, Japan) and ACDSee5.0 software (ACD Systems International Inc., Seattle, WA,USA) for image acquisition (scale bar=10 *μ*m). (**c**) Representative contour plots of bmEos on day 10. (**d** and **e**) Percentage (**d**) and total cell number (**e**) of bmEos *in vitro* bone-marrow cultures with PHPS-1 (20 *μ*M) treatment on days 4 and 8. (**f**) Representative contour plots of bmEos on day 10 with PHPS-1 (20 *μ*M) treatment on days 4 and 8. (**g** and **h**) Percentage and number of bmEos on day 10 *in vitro* bone-marrow culture with PHPS-1 (20 *μ*M) treatment on days 4 and 8. The results are expressed as means±S.E.M. (shown are three experiments performed in triplicate). (**i**) Density plots of anti-SiglecF- and AnnV-stained bmEo cells on day 10 with PHPS-1 (20 *μ*M) treatment on days 4 and 8. Numbers in quadrants indicate percentages of cells corresponding to this quadrant. (**j**) Density plots of Annexin V and PI of bmEos on day 10 with PHPS-1 treatment. Gated on SiglecF^+^ cells. (**k–m**) Analysis of the percentage of SiglecF^+^AnnV^+^, SiglecF^−^AnnV^+^ and total AnnV^+^ cells of bmEos on day 10 with the PHPS-1 treatment. The results are expressed as means±S.E.M. (shown are three experiments performed in triplicate.) n.s, not significant, *P<0.05, control group *versus* PHPS-1 group. (**n**) Schematic map of bone-marrow ablation of *shp2 in vitro*. Exon 4 of the *shp2* gene was targeted by transient *Cre* expression in bone-marrow cells from *Shp2*^*flox/flox*^ mice mediated by Cre recombinase delivered to the cells via an Ade-*Cre*-GFP adenoviral vector. (**o** and **p**) Successful deletion of *shp2* was confirmed by western blotting analysis of bmEos from *Shp2*^*flox/flox*^ mice treated with Ade-*Cre*-GFP or Ade-GFP control. The results in **p** are expressed as means±S.E.M. (shown are three experiments performed in triplicate). (**q** and **r**) Percentage and number of bmEos on day 10 *in vitro* bone-marrow culture with Ade-*Cre*-GFP treatment. The results are expressed as means±S.E.M. Shown are three experiments performed in triplicate. n.s, not significant, *P<0.05, **P<0.01, control *versus* PHPS-1 group or Ade-GFP group *versus* Ade-*Cre*-GFP group

To begin to test the function of SHP2 in eosinophil differentiation, we first analyzed the effect of phenylhydrazonopyrazolone sulfonate, PHPS-1,^25^ as a cell-permeable compound, which is highly specific for SHP2 over the closely related tyrosine phosphatases Shp1 and PTP1B, on the outgrowth of eosinophils from purified bone-marrow cells. Non-adherent mononuclear cells (NAMNCs) were first cultured for 4 days with recombinant mouse FLT3 ligand (rmFlt3-L; 100 ng/ml) and recombinant mouse stem cell factor (rmSCF; 100 ng/ml), and then cultured for 6 days with rmIL-5 (10 ng/ml) for eosinophil differentiation (Figure 1a; detailed in Materials and Methods). This induced the development of eosinophils that contained eosinophilic granules and a characteristic donut-shaped nucleus, as observed by Wright–Giemsa staining (Figure 1b). Flow cytometric analysis confirmed the development of eosinophils, as these cells were SSC^hi^ SiglecF^+^ (Figure 1c). PHPS-1 (20 *μ*M) administration together with IL-5 dramatically reduced the production of eosinophils (Figures 1d–h). However, there was no change in the production of eosinophils when PHPS-1 was given in the first 4 days before IL-5 was given (Supplementary Figures 1a–c), suggesting that the decreased eosinophil generation was not due to alterations in the numbers of very early progenitors (likely GMPs, before IL-5 stimulation to EoPs). We further detected the effect of PHPS-1 on the cell apoptosis during bone-marrow eosinophil (bmEo) differentiation *in vitro*. Analysis of the percentage and total SiglecF^+^ Annexin V^−^ cells demonstrated that there was markedly increased percentage and total viable eosinophils in the control group in comparison with PHPS-1-treated group (Figures 1i and j and Supplementary Figures 1d and e). PHPS-1 failed to have any considerable effect on the apoptosis of SiglecF^+^ cells, while increasing the apoptosis of SiglecF^−^ cells (Figures 1k and l and Supplementary Figures 1f and g), in which most cells were likely EoPs after IL-5 sitmulation. Moreover, the total Annexin V^+^ cells between the control group and the PHPS-1-treated group were similar (Figure 1m and Supplementary Figure 1h). Thus, the decreased number of bmEos by PHPS-1 may partly due to the increased apoptosis of EoPs. These data together suggest that PHPS-1 inhibits the differentiation of eosinophils without affecting their survival, while increasing the apoptosis of EoPs.

We next harvested the bone-marrow NAMNCs of *Shp2*^*flox/flox*^ mice, and infected them with Ad-*Cre*-GFP to induce *shp2* knockdown *in vitro* (Figure 1n). Western blot analysis confirmed that the level of the SHP2 protein was indeed significantly reduced in these cells (Figures 1o and p). Consistent with the effect of PHPS-1, bone-marrow NAMNCs in which *shp2* was deleted showed remarkably reduced eosinophil percentages compared with controls (Figures 1q and r).

### SHP2 is required for IL-5-induced colony formation

**Figure 2:**
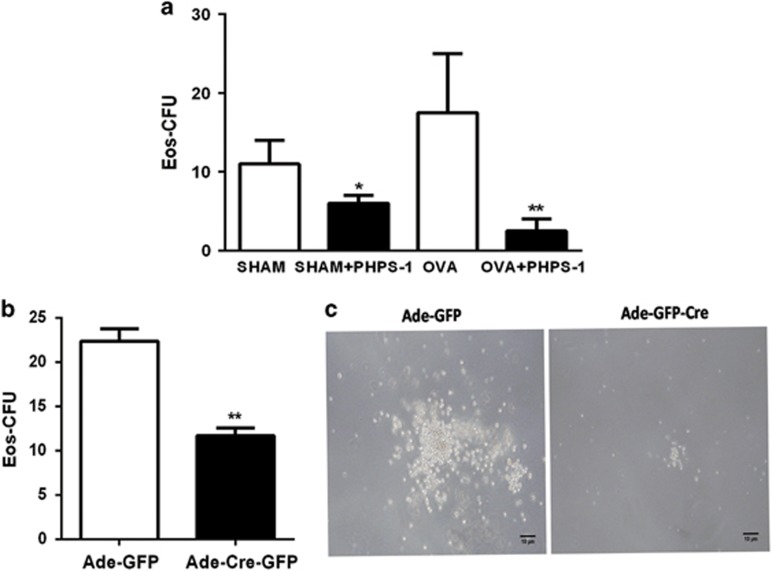
SHP2 regulates IL-5-induced colony formation. (**a**) Effects of PHPS-1 on Eos-CFU *in vitro*. Twenty-four hours after the last saline or OVA challenge, bone-marrow NAMNCs were isolated from WT and OVA mice. Cells were incubated *in vitro* at 37 °C for eosinophil colony-formation assays with IL-5 (10 ng/ml) and with the treatment of PHPS-1 (20 *μ*M) or not. Results are expressed as means±S.E.M. (*n*=6-8 mice/group). (**b** and **c**) Effect of *shp2* knockdown on Eos-CFU *in vitro*. Bone-marrow NAMNCs were isolated from *Shp2*^*flox/flox*^ mice and transduced with Ade-*Cre*-GFP or Ade-GFP, and then incubated *in vitro* at 37 °C for eosinophil colony-formation assays (scale bar=10 *μ*m). The results are expressed as means±S.E.M. Shown are three experiments performed in triplicate. *P<0.05, **P<0.01, Ade-GFP group *versus* Ade-*Cre*-GFP group

To assess the effect of SHP2 to IL-5-induced differentiation of eosinophils, we subjected cells from wild-type (WT) and allergic mice to colony-forming unit (CFU) assay using IL-5 with the treatment of PHPS-1 or not *in vitro*. The PHPS-1 group displayed a specific decrease in CFUs induced by IL-5 (Figure 2a). Furthermore, the number of eosinophil CFU (Eos-CFU) was remarkably reduced when NAMNCs were treated with PHPS-1 *in vitro*, especially those from allergic mice (Figure 2a), in which IL-5R*α* was highly expressed. Consistent with the effect of PHPS-1, bone-marrow NAMNCs in which *shp2* was deleted showed remarkably reduced numbers of Eos-CFU compared with controls (Figure 2b). Furthermore, in response to IL-5, the Eos-CFUs were smaller in NAMNCs treated with Ad-*Cre*-GFP in comparison with the Ad-GFP group (Figure 2c). These data further suggest that SHP2 indeed has a key role in the development of eosinophils.

### Genetic knockdown of *shp2* decreases eosinophil percentage in the bone marrow

**Figure 3:**
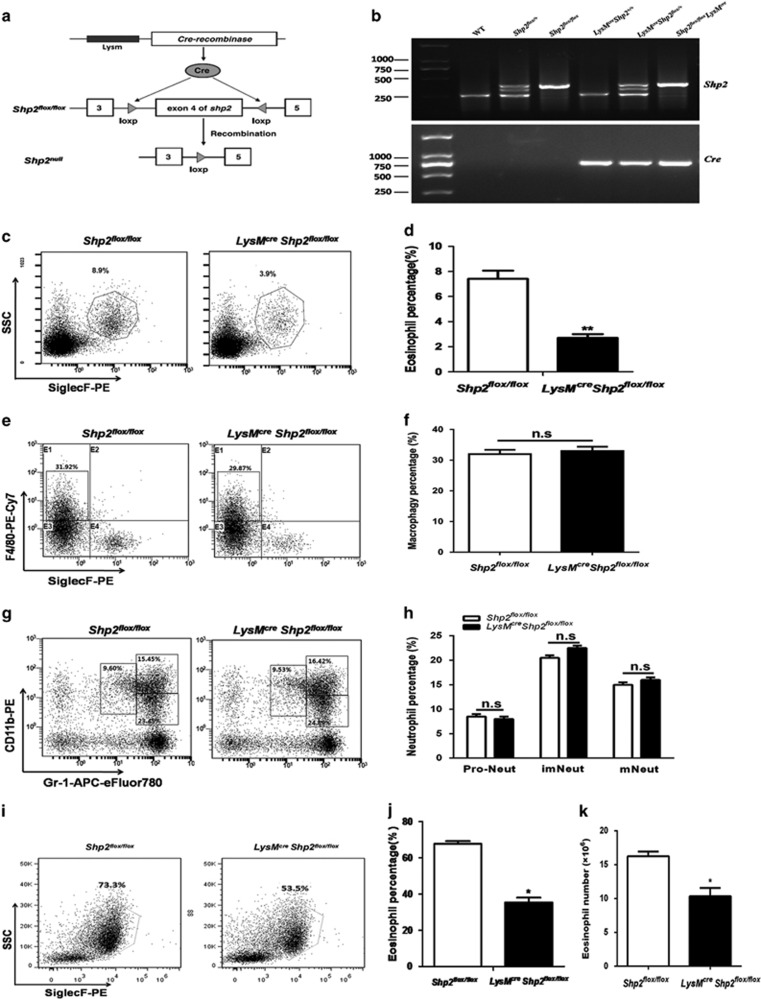
Eosinophils are decreased in bone-marrow cells of *Lysm*^*cre*^ *Shp2*^*flox/flox*^ mice. (**a**) Schematic of ablation of *shp2* in bone-marrow myeloid cells. (**b**) Genotyping performed with PCR assays using mouse tail genomic DNA. (**c** and **d**) Percentage of eosinophils in the total bone-marrow cells of *Lysm*^*cre*^ *Shp2*^*flox/flox*^ mice. Results in **d** are expressed as means±S.E.M. (*n*=6–8 mice/group). (**e** and **f**) Percentage of macrophages in the total bone-marrow cells of *Lysm*^*cre*^ *Shp2*^*flox/flox*^ mice. Results in **f** are expressed as means±S.E.M. (*n*=6–8 mice/group). (**g** and **h**) Percentage of neutrophils in the total bone-marrow cells of *Lysm*^*cre*^ *Shp2*^*flox/flox*^ mice. Results in **h** are expressed as means±S.E.M. (*n*=6–8 mice/group). (**i**) Representative contour plots of bmEos *in vitro* bone-marrow culture from *Shp2*^*flox/flox*^ and *LysM*^*cre*^*Shp2*^*flox/flox*^ mice on day 10. (**j** and **k**) Percentage and number of bmEos *in vitro* bone-marrow culture from *Shp2*^*flox/flox*^ and *Lysm*^*cre*^ *Shp2*^*flox/flox*^ mice on day 10. The results are expressed as means±S.E.M. Shown are three experiments performed in triplicate. *P<0.05, **P<0.01, *LysM*^*cre*^*Shp2*^*flox/flox*^ group *versus Shp2*^*flox/flox*^ group

To further investigate the *in vivo* and *in vitro* function of SHP2 in the bone marrow, we generated *LysM*^*cre*^*Shp2*^*flox/flox*^ mice in which *Cre* expression was induced, and then inactivated the *shp2* gene in myeloid cells (Figure 3a). Analysis of the genomic DNA from tails indicated expression of the floxed *shp2* and the *Cre* genes (Figure 3b). We first detected the base level of eosinophils in the bone marrow using flow cytometry and found that they were remarkably decreased in the *LysM*^*cre*^*Shp2*^*flox/flox*^ mice (Figures 3c and d), whereas they yielded normal numbers of macrophages (gated as SiglecF^−^ F4/80^+^; Figures 3e and f) and neutrophils (gated as Gr-1^int^ CD11b^int^, Gr-1^+^ CD11b^lo^ and Gr-1^+^ CD11b^+^, likely representing pro/mye and immature and mature neutrophils, respectively; Figures 3g and h). Furthermore, eosinophils were dramatically decreased in the bone marrow of *LysM*^*cre*^*Shp2*^*flox/flox*^ mice during the eosinophil differentiation *in vitro* (Figures 3i–k), whereas the decreased level of eosinophils was not due to alterations in the number of eosinophil progenitors (defined as Lin^−^ Sca-1^−^ CD34^+^ c-Kit^lo^ IL-5R*α*^+^; Supplementary Figure 1i). In addition, lin^−^ cells were sorted and cultured with G-CSF and PHPS-1 *in vitro* to find whether SHP2 is required in neutrophil development. Although the percentage of mature neutrophils (defined as Gr-1^+^ CD11b^+^) was decreased and that of immature neutrophils (defined as Gr-1^int^ CD11b^+^) was increased in the PHPS-1 group, the total numbers of mature and immature neutrophils were decreased significantly (Supplementary Figures 1j–l), which suggested that PHPS-1 inhibited the production of neutrophils. Collectively, these data indicate that SHP2 is required for eosinophil differentiation and can also affect on the development of neutrophils.

### SHP2 regulates IL-5-induced eosinophil differentiation via p-Erk activation

**Figure 4:**
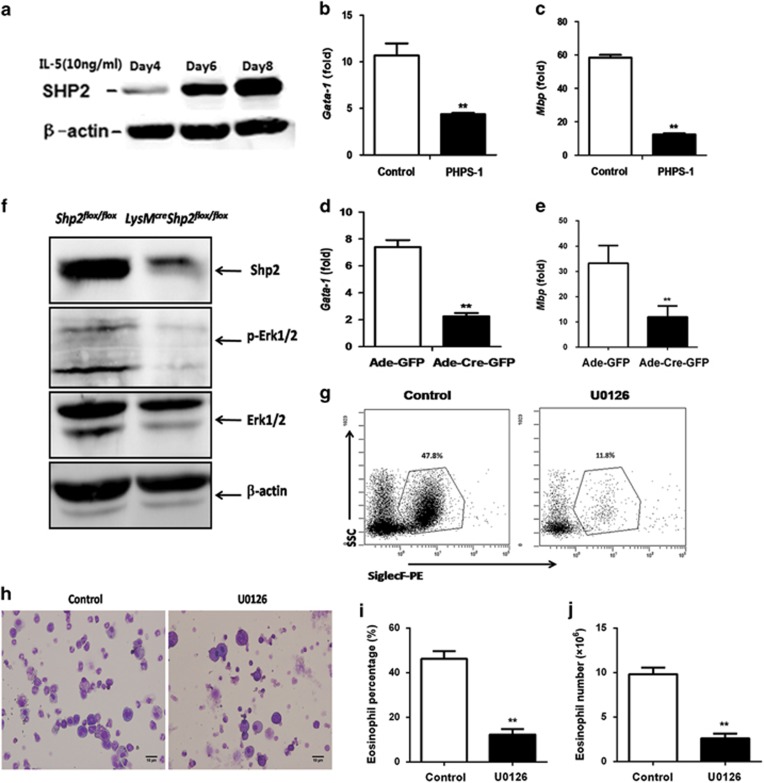
SHP2 regulates bmEo differentiation via p-Erk activation. (**a**) SHP2 protein levels during bmEo differentiation. (**b** and **c**) Effects of PHPS-1 on the mRNA levels of *Gata-1* and *Mbp* of bmEos on culture day 10. The results are expressed as means±S.E.M. Shown are three experiments performed in triplicate. (**d** and **e**) Effect of *shp2* knockdown *in vitro* by Ade-*Cre*-GFP on the mRNA level of *Gata-1* and *Mbp* of bmEos on culture day 10. The results are expressed as means±S.E.M. Shown are three experiments performed in triplicate. (**f**) The effect of SHP2 on Erk signal. BmEos were harvested from *Shp2*^*flox/flox*^ and *Lysm*^*cre*^ *Shp2*^*flox/flox*^ mice on culture day 10, and cells were lysed followed by immunoblotting with SHP2, p-Erk1/2 and Erk1/2. *β*-actin was used as a loading control. (**g**) Representative contour plots of bmEos on day 9 *in vitro* with U0126 treatment (20 *μ*M) on days 4 and 8. (**h**) Micrographs (× 400) of bmEos from the control group and the U0126 group on day 9, showing a stained Cytospin. Images were taken with an Olympus BX51 microscope (× 4/0.3 NA objective equipped with a mounted Olympus DP70 digital camera and ACDSee5.0 software for image acquisition (scale bar=10 *μ*m). (**i** and **j**) Percentage and number of bmEos on culture day 9 with U0126 (20 *μ*M) treatment on days 4 and 8. The results in **i** and **j** are expressed as means±S.E.M. Shown are two to three experiments performed in triplicate. **P<0.01, *LysM*^*cre*^*Shp2*^*flox/flox*^ group *versus Shp2*^*flox/flox*^ group or control group *versus* U0126 group

To explore the possible mechanisms of eosinophilopoiesis, we first measured the dynamic change of SHP2 and found that it was induced in a time-dependent manner during eosinophil differentiation (Figure 4a). Real-time PCR analysis also revealed that the IL-5-induced mRNA levels of *Gata-1* and *Mbp* were markedly reduced by PHPS-1 (Figures 4b and c). We also measured the level of *Gata-1* and *Mbp* mRNA on day 10 of eosinophil differentiation of *Shp2*^*flox/flox*^ mice, and found that both were significantly reduced when NAMNCs were transfected with Ad-*Cre*-GFP (Figures 4d and e). We next examined the downstream signaling events of SHP2 in the regulation of eosinophil differentiation and found that SHP2 protein levels were markedly decreased in the bmEos of *LysM*^*cre*^*Shp2*^*flox/flox*^ mice compared with *Shp2*^*flox/flox*^ mice (Figure 4f). Furthermore, knockdown of *shp2* suppressed activation of the Erk signal (Figure 4f), a common downstream kinase of IL-5 during eosinophil differentiation.^26^ Furthermore, the percentage and number of eosinophils also decreased when the bone-marrow cells were treated with 1,4-diamino-2,3-dicyano-1,4-bis[2-aminophenylthio] butadiene (U0126), a specific MAPK/ERK kinase inhibitor (Figures 4g–j). Furthermore, we detected the effect of U0126 on the apoptosis level of bmEos during differentiation. U0126 had no apparent effect on the apoptosis level of SiglecF^+^ eosinophils (Supplementary Figures 2a–d). Analysis of the percentage and total SiglecF^+^ Annexin V^−^ (AnnV^−^) cells demonstrated markedly increased percentage and total viable eosinophils in the control group in comparison with the U0126-treated group (Supplementary Figures 2e and f). Whereas U0126 increased the apoptosis of SiglecF^−^ cells (Supplementary Figures 2g and h), in which most were eosinophil progenitor cells, the decreased number of bmEos was partly due to the apoptosis of SiglecF^−^ cells. Total AnnV^+^ cells were also increased in the U0126-treated group (Supplementary Figures 2i and j). These data together suggest that U0126 inhibits the differentiation of eosinophils without affecting their survival while increasing the apoptosis of eosinophil progenitors. Moreover, the deficiency of SHP2 results in reduced activation of the Erk pathway, which then inhibits eosinophil differentiation in the bone marrow.

### Myeloid *shp2* knockdown alleviates airway inflammation and decreases AHR in a model of allergic asthma

**Figure 5:**
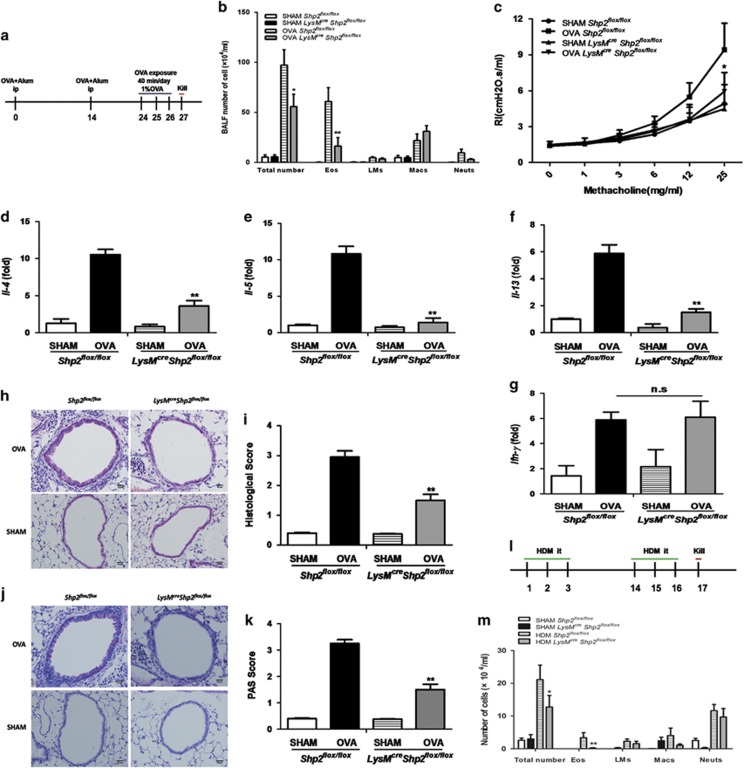
Myeloid *shp2* knockdown alleviates airway inflammation, decreases airway hyper-responsiveness and attenuates lung histopathology in allergic mice. (**a**) Schematic of OVA model. (**b**) Total numbers of BALF cells and differential counts. (Eo, eosinophil; LM, lymphomononuclear cell; Mac, macrophages; Neut, neutrophil). (**c**) Effect of myeloid *shp2* knockdown on AHR to Mch. (**d–g**) Lung *IL-4*, *IL-5*, *lL-13* and *IFN-γ* mRNA levels evaluated with qPCR. (**h–k**) Effect of myeloid *shp2* knockdown on airway inflammation (H&E staining) and goblet cell metaplasia (PAS staining) on lung tissue (× 400 magnification; scale bar=10 *μ*m). Total lung inflammation and mucus level were defined as the average of the peribronchial inflammation scores or PAS scores. (**l**) Schematic of the HDM model. (**m**) Total numbers of BALF cells and differential counts. The results are expressed as means±S.E.M. Shown are two to three experiments with six to eight mice per group per experiment. n.s., not significant, *P<0.05, **P<0.01, OVA-sensitized *Shp2*^*flox/flox*^ group *versus* OVA-sensitized *Lysm*^*cre*^ *Shp2*^*flox/flox*^ group

Regulation of eosinophil differentiation by SHP2 suggested a role for SHP2 in allergic eosinophilic airway inflammation. Therefore, we sensitized and challenged *LysM*^*cre*^*Shp2*^*flox/flox*^ and *Shp2*^*flox/flox*^ mice with chicken egg ovalbumin or house dust mite to establish two allergic models (Figures 5a and l) and explore whether conditional myeloid cell-specific *shp2* knockout inhibits lung eosinophilic inflammation. Myeloid cell-specific *shp2* knockout *LysM*^*cre*^*Shp2*^*flox/flox*^ mice showed a marked decrease in bronchoalveolar lavage fluid (BALF) inflammatory cells relative to *Shp2*^*flox/flox*^ control mice (Figures 5b and m). Moreover, AHR in *LysM*^*cre*^*Shp2*^*flox/flox*^ allergic mice was significantly reduced compared with OVA-treated *Shp2*^*flox/flox*^ controls (Figure 5c). Furthermore, myeloid cell-specific *shp2* knockdown resulted in a remarkable reduction in the mRNA levels of *IL-4*, *IL-5* and *IL-13*, whereas there was no appreciable difference in the *IFN-γ* mRNA level (Figures 5d–g). There was no obvious change in the level of serum eotaxin between OVA-treated *LysM*^*cre*^*Shp2*^*flox/flox*^ and *Shp2*^*flox/flox*^ mice (Supplementary Figure 2k). The decreased pro-inflammatory cell infiltrates in mice were further demonstrated by a corresponding alleviation in the histopathology observed in these mice as reflected quantitatively by the inflammatory and PAS scores (Figures 5h–k). These results reveal that myeloid cell-specific *shp2* knockdown in an acute allergic model inhibits airway inflammation and reduces airway responsiveness, and the mechanism is probably through inhibition of the production of eosinophils.

### PHPS-1 alleviates airway inflammation and decreases AHR in allergic mice

**Figure 6:**
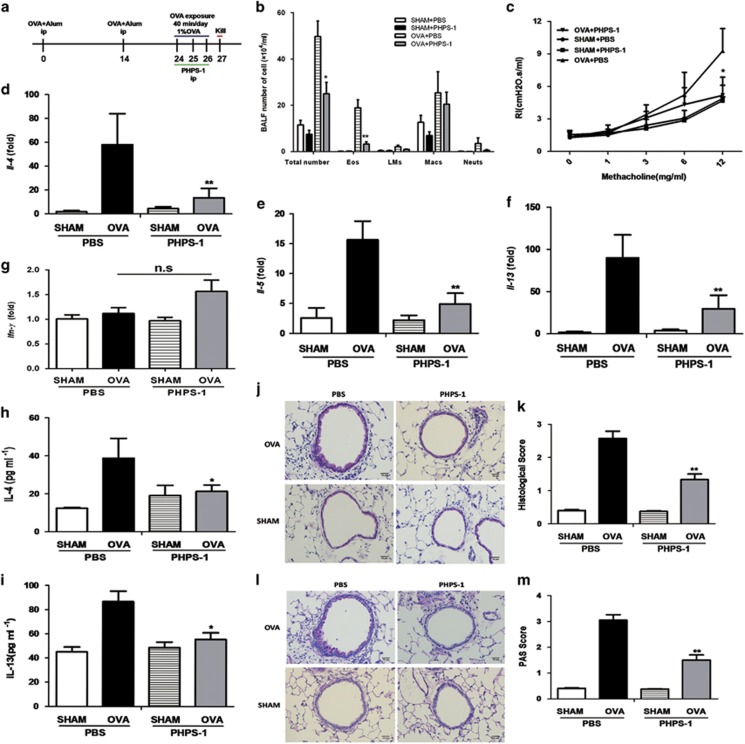
SHP2 inhibitor PHPS-1 reduces allergic inflammation. (**a**) Schematic of OVA model. (**b**) Total number of cells in BALF and differential counts. (**c**) Effect of PHPS-1 on airway hyper-responsiveness to Mch in mice 24 h after the final exposure to saline or OVA. (**d–g**) Lung *IL-4*, *IL-5*, *IL-13* and *IFN-γ* mRNA levels were evaluated with qPCR. (**h** and **i**) Lung IL-4 and IL-13 protein levels measured using ELISA. (**j–m**) Effects of PHPS-1 on airway inflammation (H&E staining) and goblet cell metaplasia (PAS staining; × 400 magnification; scale bar=10 *μ*m). Total lung inflammation and mucus levels were defined as the average of the peribronchial inflammation scores or PAS scores. Results are expressed as means±S.E.M. of two independent experiments (*n*=8 per group). n.s, not significant; *P<0.05, **P<0.01, OVA-exposed control group *versus* OVA-exposed PHPS-1 group

As myeloid *shp2* knockdown alleviates airway inflammation, we suggest that SHP2 may be a previously underappreciated therapeutic target for intervention. As such, PHPS-1 was used to explore whether it can alter allergen-induced pulmonary inflammation and become the potential therapeutic drug. We sensitized and challenged WT mice with chicken egg ovalbumin with PHPS-1 or not (Figure 6a). We quantified total and differential cell counts from BALF. As expected, PHPS-1 led to a significant reduction in total cell number, and, specifically, there was a remarkable decrease in eosinophil number in OVA/PHPS-1 mice compared with the OVA/PBS group (Figure 6b). PHPS-1 also decreased AHR in allergic mice, reducing the levels to those in the PBS-treated control group (Figure 6c). AHR to methacholine (Mch) was similar in the Control/PHPS-1 and Control/PBS groups. In addition, we assessed the effects of PHPS-1 on the mRNA levels of *IL-4*, *IL-5*, *IL-13* and *IFN-γ* in the lung. Pharmacological inhibition of SHP2 resulted in a marked reduction of the mRNA levels of *IL-4*, *IL-5* and *IL-13*; however, no detectable change was found in the *IFN-γ* mRNA level (Figures 6d–g). The protein levels of IL-4 and IL-13 in lung tissue were consistent with the mRNA levels (Figures 6h and i). The protein level of eotaxin was also reduced in the serum of OVA/PHPS-1 mice (Supplementary Figure 2l). Lung histopathology showed considerably fewer inflammatory cells in the lungs of OVA/PHPS-1 mice than OVA/PBS mice (Figures 6j and k). Periodic acid-Schiff (PAS) staining demonstrated less mucus production and fewer mucus-producing cells in the bronchioles and lungs of OVA/PHPS-1 mice than in OVA/PBS mice (Figures 6l and m). These findings reveal a potential role of PHPS-1 in protection against allergic airway inflammation.

### PHPS-1 inhibits the production of eosinophils and EoPs in allergic mice

**Figure 7:**
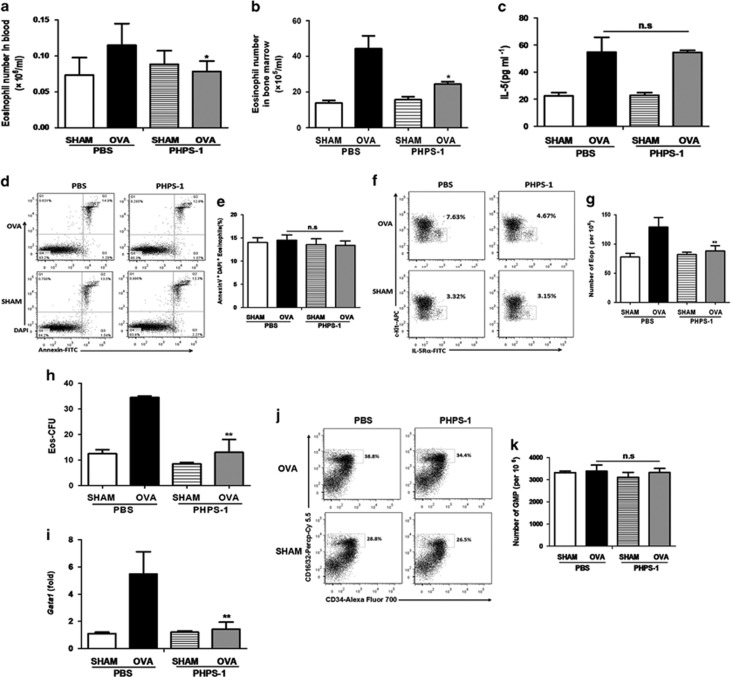
PHPS-1 inhibits the production of eosinophils and EoPs in allergic mice. (**a** and **b**) Absolute numbers of eosinophils in blood and bone marrow. (**c**) Protein levels of IL-5 in serum. (**d** and **e**) Apoptosis levels of eosinophils in bone marrow *in vivo*. Representative flow cytometry plots of eosinophils, gated on SSC^hi^ SiglecF^+^ cells (left panel). (**f**) Representative contour plots of staining for EoPs (c-kit^low^ IL-5R*α*^+^) within the Lineage^−^ CD34^+^ CD16/32^hi^ compartment of each group (percentages are shown). (**g**) Absolute numbers of EoPs in the bone marrow. (**h**) Effect of PHPS-1 on the differentiation of bone-marrow NAMNCs *ex vivo*. Bone-marrow NAMNCs were isolated from mice from each group and incubated *ex vivo* at 37 °C for colony-formation assays. (**i**) Effect of PHPS-1 on the mRNA level of *Gata-1* transcripts of bone-marrow NAMNCs in allergic mice. (**j**) Representative contour plots of staining for GMPs in the bone marrow. (**k**) Absolute numbers of GMPs in the bone marrow. Results are expressed as means±S.E.M. Shown are two to three experiments with six to eight mice per group per experiment. n.s, not significant. *P<0.05, **P<0.01, OVA-exposed control group *versus* OVA-exposed PHPS-1 group

To determine whether PHPS-1 inhibits the production of eosinophils and then eases lung inflammation, we measured the numbers of eosinophils in both blood and bone marrow. The numbers of eosinophils in blood and bone marrow decreased when allergic mice were treated with PHPS-1 (Figures 7a and b). We also analyzed the protein level of IL-5 in blood, and interestingly found no significant difference between OVA/PHPS-1 and OVA/PBS mice (Figure 7c). The apoptosis of eosinophils in the bone marrow was also similar in these two groups (Figures 7d and e). These data indicate that PHPS-1 inhibits the formation of eosinophils independent of IL-5, without influencing the apoptosis of eosinophils. Next, we evaluated the EoPs in the bone marrow and found a dramatic decrease in their number in allergic mice treated with PHPS-1 (Figures 7f and g), although there was no alteration in IL-5R*α* surface expression in eosinophil progenitors (Supplementary Figure 2m). It indicates that the decreased eosinophil numbers was possibly because of the decreased numbers of eosinophil progenitors. An *ex vivo* Eos-CFU assay further confirmed this observation, as the number of Eos-CFU from bone-marrow NAMNCs was considerably reduced in OVA/PHPS-1 mice relative to the OVA/PBS group (Figure 7h). This was supported with quantitative real-time PCR analysis of the key transcription factors in NAMNCs, as the mRNA level of *Gata-1* was inhibited in OVA/PHPS-1 mice compared with OVA/PBS mice (Figure 7i). However, the absolute number of GMPs (Lineage^−^ c-Kit^+^ CD16/32^hi^ CD34^+^) was not significantly different in OVA/PHPS-1 and OVA/PBS mice (Figures 7j and k). These results suggest that PHPS-1 inhibits the differentiation of EoPs from GMPs. These data together indicated that PHPS-1 reduces the number of EoPs and represses the expression of transcription factor *Gata-1* for eosinophil development.

## Discussion

Allergic asthma is characterized by the infiltration of eosinophils into the airway and lung tissues with progressive tissue damage.^6^ Eosinophils developed from the bone marrow and migrate into lung tissue after exposure to pulmonary allergens,^27, 28, 29^ and thus the blockade of eosinophil production should be an efficacious approach to the treatment or prevention of asthma at the source. However, to the best of our knowledge, fewer efforts have targeted the inhibition of eosinophil differentiation for the prevention of allergic airway diseases. Recently, studies have already shown that Shp2 has an important role in lung diseases,^30, 31^ especially in chronic asthma.^24^ In this study, we clearly demonstrated that SHP2 is critically involved in eosinophil differentiation. Genetic deletion of *shp2* in myeloid cells effectively reduced the number of eosinophils in the bone marrow and eventually protected mice from OVA-induced allergic airway inflammation. Pharmacological inhibition of SHP2 with PHPS-1 also markedly attenuated eosinophil differentiation *in vitro*, and decreased the numbers of eosinophils and EoPs in allergic mice. More interestingly, we also clearly demonstrated that the inhibition of SHP2 had little effect on eosinophil apoptosis. Altogether, these data strongly suggest that blockade of eosinophil differentiation, such as through SHP2 inhibition, could be a potential therapy for asthma and other consequences of eosinophilic inflammation.

The phenylhydrazonopyrazolone sulfonate PHPS-1 is a potent and cell-permeable inhibitor, which is specific for SHP2 and inhibits SHP2-dependent cellular events and downstream signaling.^25^ Recent studies^30^ further suggest that PHPS-1 inhibited the activation of Erk1/2 by CS and PHPS-1 was given 10 *μ*M *in vitro* and 3 mg/kg *in vivo* 30 min before CS exposure. We established OVA-induced allergic model with PHPS-1 according to their method with minor modification. In our work, although PHPS-1 effectively attenuated eosinophil differentiation *in vitro*, its overall protective role *in vivo* cannot rule out other possible mechanisms such as the inhibition of functions in the epithelium, neutrophils and T cells. We have recently shown that SHP2 expression is induced locally in the airway during asthma development, and genetic knockdown of shp2 in airway epithelia significantly attenuates OVA-induced airway remodeling and lung dysfunction,^24^ suggesting an important role of SHP2 in the regulation of local airway damage. Thus, the protection by PHPS-1 against allergic airway inflammation may also be due in part to its possible function in the prevention of airway damage.

Previous studies have shown that SHP2 is a positive regulator of growth-factor signaling.^17, 20^ Similarly, we also found that the SHP2 protein level was increased significantly, and this in turn positively regulated eosinophil development, depending on Erk activity. Adachi *et al.*^32^ have shown that ERK1/2 MAP kinases have an important role in eosinophil differentiation. IL-5 signaling is critical in eosinophil development and survival.^33, 34, 35^ Specific domains within the *α*c receptor of IL-5 may contain information necessary to initiate EoP proliferation and bmEo differentiation through propagation via distinct signaling pathways, resulting in a dramatic increase in the activity of SHP2 phosphatase and other downstream molecules. Feng *et al.*^18^ also demonstrated that normal SHP2 function is critical in mediating the appropriate levels of activated Erk and signal transducer and activator of transcription 3 (Stat3) necessary to maintain the proper balance of stem cell differentiation, self-renewal and apoptosis. Kano *et al.*^36^ showed that Erk activation is critical in delivering pro-apoptotic signals via Siglec-8 in IL-5-activated eosinophils. In our data, the phosphatase activity of Erk1/2 was remarkably reduced when shp2 was conditionally deleted in *LysM*^*cre*^*Shp2*^*flox/flox*^ mice during bmEo differentiation. Therefore, we assumed that SHP2 regulates eosinophil differentiation at least partially through the Erk1/2 MAPK pathway. To prove this hypothesis, a pharmacological inhibitor of Erk1/2, U0126, was used in the experiment. We found U0126 also significantly reduced the eosinophil differentiation. Thus, SHP2 has a positive role in IL-5-induced activation of the Ras-Erk kinase pathway and leads to bmEo differentiation in the bone marrow.

It might be noteworthy that PHPS-1 markedly decreased the OVA-induced EoPs while having no appreciable effects on GMPs. These data suggested that SHP2 is probably not involved in OVA-induced GMP production. Moreover, the *in vitro* study also showed that there was no change in eosinophils when PHPS-1 was treated before IL-5 was given, suggesting that the decreased eosinophil was not due to alterations in the numbers of GMPs. However, whether SHP2 directly regulates the development of EoPs from GMPs is not clear. If SHP2 is involved in EoP production from GMPs, PHPS-1 could have a direct regulatory effect on this process. Our *in vitro* study proved that the apoptosis of SiglecF^−^ cells were increased, in which most cells were likely EoPs. These data indicated that PHPS-1 increases the apoptosis of EoPs. However, SHP2 knockdown mediated by the *M-lysozyme* gene (*LysM*) should not change the SHP2 levels during the development of EoPs from GMPs. The *LysM* gene is strongly expressed in mature macrophages and myeloid cells, and is a marker of myeloid differentiation. It is progressively turned on during differentiation from myeloid precursor cells to mature eosinophils, in which it is fully active.^37, 38^ In such a case, the decreased eosinophils in the OVA-challenged *LysM*^*cre*^*Shp2*^*flox/flox*^ mice might be due to the decreased differentiation of eosinophils from EoPs in the bone marrow. Furthermore, PHPS-1 has no effect on serum IL-5 production in allergic mice, which may indicate that PHPS-1 can directly inhibit eosinophil development both *in vitro* and *in vivo*.

As the *LysM* gene is expressed in mature macrophages and myeloid cells, the next question is whether myeloid SHP2 knockdown alters the development of neutrophils or macrophages. We found that neutrophils were significantly decreased in the BALF of *LysM*^*cre*^*Shp2*^*flox/flox*^ allergic mice and OVA-challenged mice treated with PHPS-1, although there was no considerable difference in the base level of bone-marrow neutrophils. The *in vitro* neutrophil number also decreased when lineage-negative (Lin^−^) mouse marrow cells were cultured with G-CSF and PHPS-1. Jack *et al.*^39^ found that G-CSF activate STAT3 and SHP2, and potentially shifts the balance to granulopoiesis via the effects of SHP2 on regulators. These data suggest that SHP2 also has a critical role in neutrophil development. However, we found that monocytes were similar in the BALF of allergic mice either with myeloid SHP2 knockdown or with PHPS-1 treatment. Jack *et al.*^39^ have also shown that M-CSF activates ERK independently of SHP2 in monopoiesis. Thus, SHP2 may have no effects on the development of monocytes or macrophages.

The detailed mechanisms by which SHP2 regulates eosinophil differentiation remain unclear. Several lines of evidence have demonstrated unequivocally that GATA-1 has an integral role in the differentiation of myeloid progenitors to the eosinophil lineage and the regulation of eosinophil-specific genes.^40, 41, 42^ Disruption of a high-affinity double-palindromic GATA site in the mouse GATA-1 promoter results in the selective loss of the eosinophil lineage *in vivo*.^37, 38^ We showed that inhibition of SHP2 activity in the WT NAMNCs by PHPS-1, or reduction in SHP2 levels in NAMNCs from *Shp2*^*flox/flox*^ mice by Ade-*Cre* effectively decreased the expression of *Gata-1*. These data suggested the possibility that SHP2 regulates eosinophil differentiation through downregulation of *Gata-1* expression. However, these data do not rule out the possibility that SHP2 regulates eosinophil differentiation through other pathways that eventually results in decreased numbers of eosinophils, and the decreased *Gata-1* expression is just a marker of eosinophil downregulation in this case. Nonetheless, the detailed molecular pathways that mediate SHP2 function in eosinophil differentiation warrant further study.

In summary, we have demonstrated that SHP2 is a potential positive regulator of eosinophil differentiation, and inhibition of SHP2 specifically in myeloid cells eventually alleviates eosinophilic airway inflammation. Our study also represents an initial effort to demonstrate that targeting eosinophil differentiation could be an effective therapeutic approach to asthma.

## Materials and Methods

### Mice

All animal procedures conformed to the Guide for the Care and Use of Laboratory Animals and were approved by the Zhejiang University Medical Laboratory Animal Care and Use Committee. WT C57BL/6 mice were purchased from the Laboratory Animal Center of the Zhejiang University (Hangzhou, China). *Shp2*^*flox/flox*^ and *LysM*^*cre*^ mice on the C57BL/6 background were a generous gift from Dr. Gen-Sheng Fen (University of California at San Diego, CA, USA). *LysM*^*cre*^*Shp2*^*flox/flox*^ mice and littermate controls (*Shp2*^*flox/flox*^) were used for the experiments. All protocols were approved by the Ethics Committee for Animal Studies at the Zhejiang University. The primers used for gene typing were as follows: *shp2*: forward, 5′-ACGTCATGATCCGCTGTCAG-3′ reverse, 5′-ATGGGAGGGACAGTGCAGTG-3′ *Cre*: common primer, 5′-CCCAGAAATGCCAGATTACG-3′ mutant primer, 5′-CTTGGGCTGCCAGAATTTCTC-3′.

### Isolation and *ex vivo* culture of mouse bmEos

The method of *ex vivo* culture of bmEos was as described previously with slight modification.^41^ Briefly, bone-marrow NAMNC cells were cultured at 10^6^/ml in Iscove's modified Dulbecco's medium (IMDM; Invitrogen, Waltham, MA, USA) with 20% FBS (Invitrogen), 100 IU/ml penicillin and 10 mg/ml streptomycin, 2 mM glutamine, 25 mM HEPES, 1 × nonessential amino acids, 1 mM sodium pyruvate and 0.006‰ *β*-mercaptoethanol (Sigma-Aldrich, St. Louis, MO, USA) supplemented with 100 ng/ml rmSCF (PeproTech, Rocky Hill, NJ, USA) and 100 ng/ml rmFLT3-L (PeproTech) from days 0 to 4. On day 4, the medium was replaced with medium containing 10 ng/ml rmIL-5 (R&D Systems, Minneapolis, MN, USA). On day 8, the cells were provided with fresh medium supplemented with rmIL-5. In most experiments, 20 *μ*M PHPS-1 (Sigma-Aldrich) was treated on days 4 and 8 when the medium was replaced. While in some experiments 20 *μ*M PHPS-1 (Sigma-Aldrich) was treated on day 0 and washed off on day 4 when the medium was replaced. U0126 (20 *μ*M; Cell Signaling Technology, Danvers, MA, USA) was treated on days 4 and 8 when the medium was replaced. Cells were enumerated and harvested for analysis of the mRNA levels of *Gata-1* and *Mbp*. Apoptosis was detected and cells were stained with SiglecF for anylysis of eosinophils. Cells were lysed and analyzed by western blotting with polyclonal ERK1/2, p-ERK1/2, SHP2 and *β*-actin. Antibodies against ERK and p-ERK were from Cell Signaling Technology, and antibodies against SHP2 and *β*-actin were from Santa Cruz Biotechnology (Dallas, TX, USA). Proteins were visualized with fluorescent antibodies. Fluorescence signals were captured with Odyssey Imaging (Li-COR Biosciences, Lincoln, NE, USA) system, and quantified with Li-COR Image Studio. All blot images shown in the figures were representatives from at least three independent experiments.

### Isolation and *ex vivo* culture of mouse bone-marrow-derived neutrophils

The Lin^−^ cells were collected by BD Influx and cultured with 100 ng/ml rmG-CSF for 10 days with PHPS-1 treatment (20 *μ*M). Neutrophils were analyzed on culture day 10. For analysis of neutrophils, cells were stained with APC-eFluor780-conjugated Gr-1 (eBioscience, San Diego, CA, USA) and PE-conjugated CD11b (eBioscience) in the presence of anti-CD16/32 block (2.4 G2; BD Pharmingen, San Jose, CA, USA). Gr-1^lo^/CD11b^+^ and Gr-1^hi^/CD11b^+^ subsets defined immature and mature neutrophil populations, respectively.^43^

### Shp2 conditional deletion in the bone marrow by adenovirus transfection *in vitro*

Mouse bone-marrow NAMNCs were collected as described previously;^44^ NAMNCs from *Shp2*^*flox/flox*^ mice were transduced with Ad-*Cre*-GFP and Ad-GFP at a multiplicity of infection of 20 for 6 h and further cultured with rmIL-5 (10 ng/ml) for the assay of bmEo CFUs. For bmEo differentiation, NAMNCs from *Shp2*^*flox/flox*^ mice were first cultured with 100 ng/ml SCF and 100 ng/ml FLT3-L for 4 days and then transduced with Ad-*Cre*-GFP and Ad-GFP at a multiplicity of infection of 20 for 6 h and further cultured with rmIL-5 (10 ng/ml) for the assay of bmEos. On day 8, the efficiency of *shp2* deletion was determined.

### Assay for bone-marrow colony-forming units

A total of 2 × 10^4^ bone-marrow NAMNCs was supplemented with IL-5 (10 ng/ml) and cultured in IMDM supplemented with 0.9% methylcellulose (Stem Cell), 20% fetal bovine serum (GIBCO BRL, Grand Island, NY, USA),1% penicillin–streptomycin, 2 mM L-glutamine and 0.006% *β*-mercaptoethanol in 3.5-cm dishes (Corning, Corning, NY, USA) at 37 °C under 5% CO_2_. After 14 days, colonies (>50 cells) were counted using an inverted microscope and classified using morphological and histological criteria. Eos-CFU was also determined by expression of SiglecF (>90%).^45^

### Flow cytometry

For analysis of eosinophils in the bone marrow using flow cytometry, cells were stained with PE-conjugated SiglecF (BD Pharmingen) in the presence of anti-CD16/32 block (2.4 G2; BD Pharmingen). For the identification of EoPs (Lineage^−^ CD34^+^ CD16/32^hi^ c-kit^low^ IL-5 Rα^+^) and GMPs (Lineage^−^ c-Kit^+^ CD16/32^hi^ CD34^+^), the antibodies used were FITC-conjugated anti-IL-5R*α* chain (H7; BD Pharmingen), Alexa Fluor700-conjugated anti-CD34 (RAM34; eBioscience), APC-conjugated anti-c-Kit (2B8; eBioscience), PE-Cy5-conjugated anti-Sca-1 (D7; Biolegend, San Diego, CA, USA) and Perp-Cy5.5-conjugated anti-CD16/32 monoclonal antibodies (eBioscience), as well as a lineage cocktail (CD4 (RM4-5; Biolegend), CD8a (53-6.7; Biolegend), CD11b (M1/70; Biolegend), CD45R/B220 (RA3-6B2; Biolegend), Gr-1 (RB6-8C5; Biolegend) and TER-119/Erythroid cells, followed by streptavidin-APC-Cy7 (BD Pharmingen). Dead cells were excluded by using DAPI. Cell staining was determined with a BD LSRFortessa cell analyzer. Flow data were analyzed and plotted with the FlowJo software (Treestar Inc.). Apoptosis was detected using an Annexin V apoptosis detecion kit (eBioscience) according to the manufacturer's instructions.

For *ex vivo* lin^−^ cell cultures, bone-marrow cells from WT mice were stained with a lineage cocktail (CD4 (RM4-5; Biolegend), CD8a (53-6.7; Biolegend), CD11b (M1/70; Biolegend), CD45R/B220 (RA3-6B2; Biolegend), Gr-1 (RB6-8C5; Biolegend) and TER-119/Erythroid cells), followed by streptavidin-APC-Cy7 (BD Pharmingen). The Lin^−^ cells were collected by fluorescence-activated cell sorting (BD Influx, San Jose, CA, USA) and cultured in IMDM/FBS containing 100 ng/ml recombinant mouse G-CSF (Peprotech) for 10 days with PHPS-1 treatment (20 *μ*M). Neutrophil populations were then analyzed using flow cytometry on culture day 10.

### Ovalbumin-induced allergic airway inflammation model

Mice were intraperitoneally (i.p.) sensitized on days 0 and 14 with OVA or saline. On days 24, 25 and 26, mice were challenged with 1.5% OVA or saline for 40 min. The mice of SHAM/PBS and OVA/PBS groups were injected i.p with saline 1 h before each saline or OVA challenge, while the mice of SHAM/PHPS-1 and OVA/PHPS-1 groups were injected i.p with 5 mg/kg PHPS-1 1 h before each saline or OVA challenge. Twenty-four hours after the final OVA or saline challenge, airway responsiveness was measured using Buxco FinePointe as previously described.^46^ Mice were killed 24 h after the last exposure for BALF, blood, tissues and bone marrows. Eosinophils, EoPs and GMPs were detected in the bone marrow using flow cytometry. Lungs were fixed and stained with hematoxylin and eosin (H&E) or PAS to demonstrate the general morphology and the presence of mucin within goblet cells. Slides were digitalized with an Olympus BX51 microscope (4/0.3 NA objective) equipped with an Olympus DP70 digital camera and ACDSee5.0 software for image acquisition. The concentrations of IL-4, IL-5, IL-13 and eotaxin were determined using ELISA as per the manufacturer's instructions (R&D Systems, Minneapolis, MN, USA).

### Isolation of RNA and quantitative real-time PCR

Cells from various tissues were suspended in TRIzol reagent (Takara) and extraction proceeded as per the manufacturer's instructions. The PCR primers were from Shanghai Bioengineering (Shanghai, China). RNA was reverse-transcribed using a PrimeScript TM RT-PCR kit (Takara, Kusatsu, Shiga, Japan) and cDNA was subjected to real-time PCR with SYBR Premix Ex TaqTM (Perfect Real Time; Takara). Real-time PCR cycling was carried out on a 7500 Real-Time PCR System (Applied Biosystems, Carlsbad, CA, USA). The primers used were as follows: *Il-4*: forward, 5′-GGTCTCAACCCCCAGCTAGT-3′ reverse, 5′-GCCGATGATCTCTCTCAAGTGAT-3′ *Il-5*: forward, 5′-CTCTGTTGACAAGCAATGAGACG-3′ reverse, 5′-TCTTCAGTATGTCTAGCCCCTG-3′ *Il-13*: forward, 5′- CAGCCTCCCCGATACCAAAAT-3′ reverse, 5′-GCGAAACAGTTGCTTTGTGTAG-3′ *Ifn-γ*: forward, 5′- CCTGCAGAGCCAGATTATCTC-3′ reverse, 5′-CCTTTTTCGCCTTGCTGTTGC-3′ *Gata-1*: forward, 5′-TATGGCAAGACGGCACTCTAC-3′ reverse, 5′-GGTGTCCAAGAACGTGTTGTT-3′ *Mbp*: forward, 5′-GCAAACGCTTTCGATGGGTTG-3′ reverse, 5′-ACACAGTGAGATAGACGCCAG-3′ *β-actin*: forward, 5′-AGAGGGAAATCGTGCGTGAC-3′ reverse, 5′-CAATAGTGATGACCTGGCCGT-3′. The mRNA levels were calculated using the comparative parameter threshold cycle (*C*t) and normalized to *β*-actin.

### HDM-induced allergic airway inflammation model

Mice were intratracheally (i.t.) sensitized on days 1, 2 and 3 with 50 *μ*g house hust mite (HDM) or saline. On days 14, 15 and 16, mice were challenged with 50 *μ*g HDM or saline. Mice were killed 24 h after the last exposure to HDM or saline for the measurement of BALF cellularity.

### Statistical analysis

Data were analyzed by ANOVA followed by Tukey's *post hoc* test using GraphPad Prism 5 (GraphPad Software, Inc, La Jolla, CA, USA). Alternatively, several experiments were analyzed with Student's *t*-test. Data are expressed as mean±S.E.M. Differences between the mean values were considered significant when *P*<0.05.

## Abbreviations

GMP: granulocyte/monocyte progenitor
EoP: eosinophil lineage-committed progenitor
ES: embryonic stem
IL-5*α*cR: IL-5 receptor
PHPS-1: phenylhydrazonopyrazolone sulfonate
CFU: colony-forming unit
Eos-CFU: eosinophil colony-forming unit
U0126: 1,4-diamino-2,3-dicyano-1,4-bis[2-aminophenylthio] butadiene
AHR: airway hyper-responsiveness
BALF: bronchoalveolar lavage fluid
Mch: methacholine
PAS: Periodic acid-Schiff
NAMNC: non-adherent mononuclear cell
*LysM*: *M-lysozyme* gene
STAT3: signal transducer and activator of transcription 3
WT: wild type
rmSCF: recombinant mouse stem cell factor
rmFLT3-L: recombinant mouse FLT3 ligand
Lin-: lineage-negative
i.p.: intraperitoneally
H&E: hematoxylin and eosin
Ct: threshold cycle
i.t.: intratracheally
HDM: house hust mite
bmEo: bone-marrow eosinophil
IMDM: Iscove's modified Dulbecco's medium
AnnV: Annexin V
Eo: eosinophil
LM: lymphomononuclear cell
Mac: macrophage
Neut: neutrophil
