# Supplementary material for: Eosinophil differentiation in the bone marrow is promoted by protein tyrosine phosphatase SHP2
Source: Cell Death Dis. 2016 Apr 7;7(4):e2175–. doi: 10.1038/cddis.2016.74 (PMC4855658; doi:10.1038/cddis.2016.74)
Supplement: Supplementary Information [file cddis201674x1.docx]

**Supplementary information**

**The supplemental data includes 25 supplemental figures with legends.**

**Supplementary figure legends**

**Figure S1 (a, b, c)** Representative contour plots of SiglecF expression (a), and percentage (b) and number (c) of bmEos in *vitro* culture. Bone marrow NAMNCs were incubated with PHPS-1(20 μM) treatment from day 0 to day 4 before IL-5 was given, and bmEos was analyzed on day 10 by flow cytometry. The results are expressed as means ±S.E.M. Shown are 3 experiments performed in triplicate. There was no alteration in eosinophil population when PHPS-1 was treated from day 0 to day 4 before IL-5 was given. **(d, e)** Analysis of the percentage and total cell counts of SiglecF^+^AnnV^−^ cells on day 10 with PHPS-1 (20 μM) treatment on day 4 and day 8. **(f, g, h)** Analysis of total cell counts of SiglecF^+^AnnV^+^, SiglecF^-^AnnV^+^ and total AnnV^+^ cells on day 10 with PHPS-1 (20 μM) treatment on day 4 and day 8. **(i)** Assessment of EoPs in the bone marrow of *Shp2^flox/flox^* and *LysM^cre^Shp2^flox/flox^* mice. The decreased level of eosinophils was not due to alterations in the number of eosinophil progenitors. **(j)** Representative contour plots of neutrophils *in* *vitro* lin^-^ cell culture with PHPS-1 (20 μM) treatment. Cells were analyzed on culture day 10 by flow cytometry. **(k, l)** Percentage and number of Gr-1^lo^/CD11b^+^ cells and Gr-1^hi^/CD11b^+^ cells *in* *vitro* lin^-^ cell culture with PHPS-1 (20 μM) treatment. Cells were analyzed on culture day 10. The results are expressed as means ±S.E.M. (Shown are 3 experiments performed in triplicate.). *P <0.05, control group *versus* PHPS-1 group.

**Figures S2 (a)** Density plots of anti–SiglecF– and Annexin V–stained bmEos on day 9 with U0126 (20 μM) treatment on day 4 and day 8. Numbers in quadrants indicate percentages of cells corresponding to this quadrant. (**b**) Density plots of Annexin V and PI of bmEos on day 9 with U0126 (20 μM) treatment on day 4 and day 8. Gated on SiglecF^+^ cells. (**c, d, e, f, g, h, i, j**) Analysis of the percentage and total cell counts of SiglecF^+^AnnV^+^, SiglecF^+^AnnV^−^, SiglecF^-^AnnV^+^ and total AnnV^+^ cells. Cells were treated with U0126 (20 μM) on day 4 and day 8 and harvested on day 9. The results are expressed as means ±S.E.M. (Shown are 3 experiments performed in triplicate.) n.s, not significant, **P <0.01, control group *versus* U0126 group. (**k)** The eotaxin level in serum between *LysM^cre^Shp2^flox/flox^* group and *Shp2^flox/flox^* group. The level of serum eotaxin was no obvious change in *LysM^cre^Shp2^flox/flox^* mice. The results are expressed as means ±S.E.M. (n = 6-8 mice/group). n.s, not significant, *LysM^cre^Shp2^flox/flox^* group *versus Shp2^flox/flox^* group. (**l)** The eotaxin level in serum. The level of serum eotaxin was reduced in OVA/PHPS-1 mice. The results are expressed as means ±S.E.M. (n = 6-8 mice/group). * P <0.05, OVA-exposed control group *versus* OVA-exposed PHPS-1 group. (**m)** Assessment of IL-5Rα MFI in EoPs. There was no alteration in IL-5Rα surface expression in eosinophil progenitors. The results are expressed as means ±S.E.M. (n = 6-8 mice/group). Shown are 3 experiments performed in triplicate. *P <0.05, control group *versus* PHPS-1 group.
